# Autoimmunity and Genetic Syndromes: A Focus on Down Syndrome

**DOI:** 10.3390/genes12020268

**Published:** 2021-02-13

**Authors:** Marta Ferrari, Stefano Stagi

**Affiliations:** Health Sciences Department, University of Florence, Anna Meyer Children’s University Hospital, Viale Pieraccini 24, 50139 Florence, Italy; stefano.stagi@unifi.it

**Keywords:** Down syndrome, autoimmunity, immune dysregulation

## Abstract

Within immune system-related diseases, autoimmunity has always represented a field of great interest, although many aspects remain poorly understood even today. Genetic syndromes associated with immunity disorders are common and represent an interesting model for a better understanding of the underlying mechanism of autoimmunity predisposition. Among these conditions, Down syndrome (DS) certainly deserves special attention as it represents the most common genetic syndrome associated with immune dysregulation, involving both innate and adaptive immunity. Autoimmunity represents a well-known complication of DS: it is estimated that people affected by this disease present a risk four to six times higher than the normal population to develop autoimmune diseases such as celiac disease, type 1 diabetes mellitus, and hypo- or hyperthyroidism. Several factors have been considered as possible etiology, including genetic and epigenetic modifications and immune dysregulation. In times in which the life expectancy of people with DS has been extremely prolonged, thanks to improvements in the diagnosis and treatment of congenital heart disease and infectious complications, knowledge of the mechanisms and proper management of autoimmune diseases within this syndrome has become essential. In this short review, we aim to report the current literature regarding the genetic, immune, and environmental factors that have been proposed as the possible underlying mechanism of autoimmunity in individuals with DS, with the intent to provide insight for a comprehensive understanding of these diseases in genetic syndromes.

## 1. Introduction

Autoimmunity commonly features many well-known genetic conditions, such as Turner syndrome (TS), Trisomy 21 or Down syndrome (DS), and 22q11.2 deletion syndrome (22q11.2DS) [1]. The susceptibility toward this disorder has been recently investigated for many other genetic syndromes, such as Kabuki, Noonan, and Klinefelter [2,3,4,5]. In these patients, virtually all autoimmune disorders have been described, in particular thyroid disorders and celiac disease, in addition to alopecia, vitiligo, type 1 diabetes, juvenile idiopathic arthritis, and systemic lupus erythematosus (SLE). Several mechanisms have been attributed to the etiology of autoimmunity, including genetic or acquired defects of immune regulatory pathways, impaired apoptosis, and molecular mimicry to viral or bacterial antigens [5]. Although the exact mechanisms underlying autoimmune diseases are still elusive, it appears as though a pathogenic inflammatory response by self-antigen-specific T cells is frequently involved [1]. Given the common association between autoimmunity and chromosomal aberrations, many efforts have been made with the intent of identifying possible underlying mechanisms and perhaps also provide insights for a better understanding of other clinical aspects related to genetic syndromes. Among the many genetic alterations, DS was one of the first to be studied in relation to autoimmunity. In fact, as early as 1969, an association between DS (at the time defined as Mongolism) and autoimmune thyroiditis was observed [6]. Since that time, much progress has been made in defining the main features of this syndrome and associated autoimmune complications; however, many questions still need to be answered. In this short review, we aim to report the current literature regarding mechanisms underlying autoimmunity and its main clinical outcomes, providing, where possible, recommendations for early identification, follow up, and monitoring of these disorders in DS.

## 2. Down Syndrome

Down syndrome (DS) is the most frequent aneuploidy (1:700 live births) and results from the triplication of human chromosome 21. Among the currently known genetic syndromes, DS represents the one most frequently associated with immune dysregulation [7]. While improved care for congenital heart disease has decreased mortality and morbidity, complications related to immune-mediated diseases continue to limit the life expectancy in DS patients by increasing rates of infections, hematological malignancies, and autoimmunity. However, the mechanisms underlying immune disorders are still poorly understood and have alternatively been interpreted as a consequence of precocious aging or an intrinsic defect of the immune system [8]. Genetic and molecular analysis of chromosome 21 has provided in the past few years interesting insights; several genes involved in immune system regulation are encoded on chromosome 21, including four of the six interferon (IFN) receptor subunits, which serves as receptors for both type III IFN ligands and the cytokines IL-10, IL-22, and IL-26 [9], and epigenetic-related genes, such as autoimmune regulator (*AIRE*) gene. More recently, some studies have focused on the possible role played by microRNAs (miRNAs) encoded on chromosome 21 and well represented in DS because of the extra chromosome. miRNAs are endogenous small non-protein-coding RNAs acting as negative regulators of target gene expression with observed potential as modulating transcriptome in physiological and pathological scenarios. Many of these have been extensively studied in relation to cardiac development, neurocognitive alterations, and hematopoietic tumors. Interestingly, some of these, miR-99a, let-7c, miR-125b-2, miR-155, and miR-802 expressed on chromosome 21, seem to down-regulate the expression of key innate immune regulatory and anti-inflammatory genes in DS [10], for example, complement factor H mRNA and some Toll-like receptors (TLRs), both important in initiating chronic inflammation and autoimmunity [7,9]. A recent study by Farroni et al. has also demonstrated with in vivo and in vitro analysis that miR-125 and miR-155 are overexpressed in B cells of individuals with DS and proposed this alteration as a possible cause of the well-known immunodeficiency that characterizes the syndrome [11]. How and in what way the altered expression of these genes and miRNAs may contribute to susceptibility to autoimmunity is yet to be defined. However, the analysis of immune alterations represents a good starting point for a better definition of these autoimmune clinical conditions [7,10].

## 3. Down Syndrome: Immune Dysregulation 

Several alterations to the innate and adaptive immune response components have been considered as possible candidates in explaining the observed predisposition of DS patients to autoimmune diseases.

DS has been considered for years as a model of primary immunodeficiency disorder characterized by a fundamental defect in the differentiation of B cells leading to a significant decrease in switched memory B cells [12]. Studies performed on peripheral blood mononuclear cells (PBMCs) have observed that proportions of B cell populations resemble common variable immunodeficiency (CVID) patterns, with a decrease in switched memory B cells and an increased number of likely autoreactive CD21 B cells [8]. More interestingly, individuals with DS show a specific serum immunoglobulin pattern with a progressive increase in total IgG in the first year of life, followed by a substantial normal value for the rest of their life; IgA levels are frequently normal, in contrast to decreased IgM and IgE levels. Therefore, in contrast to others, immunodeficiency patients with DS show increased levels of IgG, with a demonstrated protective antibody response to pneumococcal and tetanus immunizations. However, it seems that compared to healthy subjects, the antibody levels of such patients in response to vaccination are more reduced and tend to decline more rapidly over time [13]. 

Concerning defects in the adaptive system in, perhaps, the first and best-studied link between DS and immune system is the one concerning abnormal T cell function [14]. 

More specifically, T lymphocyte impairment starts at the very beginning of the cell maturation phase, involving not only thymic architecture but also defects of thymocyte and naïve T cell development as well as altered expansion of memory T cells [8]. 

Individuals with DS show abnormal thymic architecture characterized by hypoplasia, cortical atrophy, and increase in the size of the medullary compartment [12,14,15]; thymocytes are less represented and forced to an accelerated maturation that can alter the normal cross-talk between epithelial cell and T reg cell, both essential mechanisms for the establishment of central tolerance. Studies performed on peripheral blood have demonstrated that people with DS display a reduced number of naïve helper and cytotoxic T lymphocytes as well as T cell receptor excision circles (TRECs), which represent an indirect parameter of T cell turnover. Interestingly, these alterations occur early in childhood, anticipating the age-related involution process that comes after puberty, and could be interpreted as an early senescence of the immune system in DS patients [16,17,18].

In addition, the thymus of DS patients presents an altered expression of a large number of pivotal genes that can represent potential candidates for explaining susceptibility to autoimmunity. Among these, the already mentioned *AIRE* gene represents one of the most interesting. *AIRE* is a transcriptional factor that regulates the expression of various tissue-restricted antigens (TRAs) in thymic medullary epithelial cells, a critical mechanism for the negative selection of T cell repertoire emerging from the thymus in which self-reactive thymocytes with excessive affinity to TRAs are eliminated in the thymus before reach the periphery. *AIRE* also plays important roles in controlling antigen presentation and chemokine production and maturation. Even though studies investigating the degree of expression of this gene in thymus cells have shown mixed results [19,20], it appears clear that the final outcome is a less accurate regulation of the thymic process. An altered *AIRE* expression could, therefore, influence the thymic selection of T lymphocytes in these patients and together with thymic abnormalities lead to a greater propensity to develop autoimmune conditions [12,21,22]. Not all DS patients present autoimmune disorders, although most show reduced *AIRE* expression, suggesting that a pathogenic threshold may be influenced by other factors [19].

The problem does not only concern the reduced number of cells present and their early senescence but is also linked to an altered functionality. A recent study by Araya et al. [16] has demonstrated that CD8+ T cells from individuals with DS overproduce cytokines tied to autoimmunity and CD4+ T cells display a polarized state toward increased production of IL-17A, with elevated plasma levels of other IL-17 subunits. The role of IL-17 in the pathogenesis of inflammatory and autoimmune disease is well known, particularly in autoimmune diseases characterized by high levels of type I IFN signaling, such as in systemic lupus erythematosus and dermatomyositis. It has been hypothesized that type I IFN and IL-17 act in concert to sustain and amplify autoimmune and inflammatory responses, making them a dangerous combination involved in the pathogenesis of autoimmune diseases [16].

Another important actor involved in modulating the intensity and targets of the immune response is represented by T regulatory cells (Tregs). Several studies have been looking for a relation between Treg function and autoimmunity predisposition in people with DS, showing that, compared with healthy subjects, people with DS do not show significant phenotypic differences or impaired functional capacity. Nevertheless, both CD8+ and CD4+ T cells seem to be more resistant to Treg-mediated suppression. Resistance to suppression has previously been reported in different autoimmune diseases, such as type I diabetes and systemic lupus erythematosus (SLE). Although the mechanisms of DS are still unknown, it is likely that continued exposure to cytokines such as TNF-α overproduced by CD8+ and CD4+ after stimulation may play a role in inducing T cell resistance [10,16]. In addition, some miRNAs (miR-99a, let-7c, miR-125b-2, miR-155, and miR-802) overexpressed in the brains and hearts of DS individuals have probably a role in immune regulation. In particular, miR-155, miR-125-b, and let-7c are involved in the regulation of Treg development and macrophage responses to different stimuli [19].

As mentioned before, DS people display extensive immune dysregulation, which affects all the different fields of immune response. Since the first studies carried out on DS patients, innate immunity has been considered in relation to the increased susceptibility toward infections. In recent years, however, several studies have focused on analyzing the possible role of innate immunity in susceptibility to autoimmune diseases, with different results. On this topic, one of the most fascinating fields concerns the studies on Toll-like receptors (TLRs) and interferon (IFN) activity. 

TLRs are integral plasma membrane proteins that initiate a signaling cascade following a broad range of endogenous and exogenous stimuli, robustly activating the innate immune system. Accurate regulation of TLR pathways is crucial not only for protection against infections but also for prevention of damages deriving from an excess of cytokine production and the consequent autoimmunity that characterizes individuals with DS [23]. 

Within the big TLR family, TLR-2 plays a pivotal role in detecting bacterial infection because of its capacity of binding different signal molecules, such as pathogen-associated molecular patterns (PAMPs), damage-associated molecular patterns (DAMPs), and lipoproteins located on Gram-positive membranes. Furthermore, an increased TLR-2 expression has been reported on the monocyte subsets of patients affected with different autoimmune diseases, such as active rheumatoid arthritis, celiac disease (CD), and autoimmune thyroid disease. Emphasizing the involvement of TLR-2, Huggard et al. [23] have recently demonstrated increased TLR2 expression on neutrophils and monocyte subsets in children with DS compared to healthy controls. In addition, an impaired neutrophil chemotaxis and abnormal natural killer (NK) function are frequently found in children with DS [12]. Although further studies are needed, taken together this evidence may represent a first step toward defining the role of the innate immune system as a possible mediator in the pathogenesis of autoimmunity in these patients [23]. 

Cytokines also deserve particular attention, not only because of their well-studied biology within the context of autoimmune disease and chronic inflammation, but also as a possible mechanism of damage in inflammatory response, given that many studies have tried to investigate the possible role of these molecules and suggest that people with DS demonstrate an incremented level of both pro- (IL-2, IL-1, IL-6, TNF-α) and anti-inflammatory cytokines (IL-10, IFN-γ) at baseline and after stimulation [24,25,26]. 

In particular, a growing body of literature highlights increasing interest in a possible role for interferons (IFN). IFN are cytokines involved in different aspects of the immune system, from antiviral activity to protection against a variety of neoplastic and inflammatory conditions. By inducing the intracellular activation of the JAK/STAT pathways, type 1 IFNs are responsible for different key aspects in the pathology of various autoimmune diseases, and there are several supporting findings demonstrating a state of IFN hyperactivation also in individuals with DS. More specifically, receptors for type 1 IFN, IFN-α, and IFN-γ are encoded on chromosome 21 and, therefore, the presence of an excess copy might contribute to an increase in the number of available receptors [9]. In addition, it has been observed that both immune and non-immune cells from DS patients display an overexpression of these receptors on the cell surface, with hypersensitivity toward IFN stimulation [14,15,17].

Although the pathological mechanism linking IFN hyperactivation to the development of DS clinical hallmarks remains partly unclear, some interesting insights might come from a recent study by Tuttle et al., where they observed IFN hyperactivation in a mouse model of DS. In the same article, they also provide evidence that JAK-1 inhibition may represent a potential therapeutic target to reverse the toxic consequences of IFN chronic activation [27]. 

Interestingly, studies on diseases of IFN signaling may provide insights into the excessive inflammation observed in COVID-19 infections: although IFN hyperactivity may be considered as beneficial by boosting the antiviral response during the first contact, studies of the SARS-CoV-1 and the Middle East Respiratory Syndrome CoV (MERS-CoV) have revealed that their genomes encode an unusual number of proteins that dampen or neutralize the IFN signaling cascade. Furthermore, DS increases the likelihood of developing a stronger and more prolonged cytokine storm and, therefore, DS patients should be considered a high-risk population for complications driven by SARS-CoV-2-induced hyperinflammation [24,28].

Therefore, the immune dysregulation observed in DS is clinically relevant in two different ways. Firstly, because of the previously discussed immune alterations, patients are at greater risk of developing infectious diseases in terms of more severe infections and poorer outcomes. In addition, they are more likely to be admitted into a hospital, have an increased length of stay due to respiratory tract infection, and have a greater chance of requiring ventilatory support and intensive care, with increased risk to develop sepsis. Studies focused on respiratory infections as well as cohort studies linking respiratory syncytial virus and the H1N1 2009 pandemic influenza with higher incidences of hospitalization demonstrate this. However, there are limited data concerning other types of infections [8,12,14,29]. In addition, it should be considered that recurrent infections, especially if viral, represent a predisposing factor to the development of autoimmunity [30]. 

Secondly, these results suggest that even in the absence of a clinical manifestation of autoimmunity, DS patients’ immune systems display common phenotypic alterations observed in autoimmune and autoinflammatory conditions [16]. The problem becomes more challenging in adults where these changes are similar to those associated with the chronic state of low-grade inflammation observed during typical aging and in various inflammatory and autoimmune states in the general population [15,20]. However, in which way and to what extent hyperactive IFN signaling, inflammatory cytokines, and specific immune cell types can cause the autoimmunity disease spectrum in DS remains to be defined.

**Figure 1:**
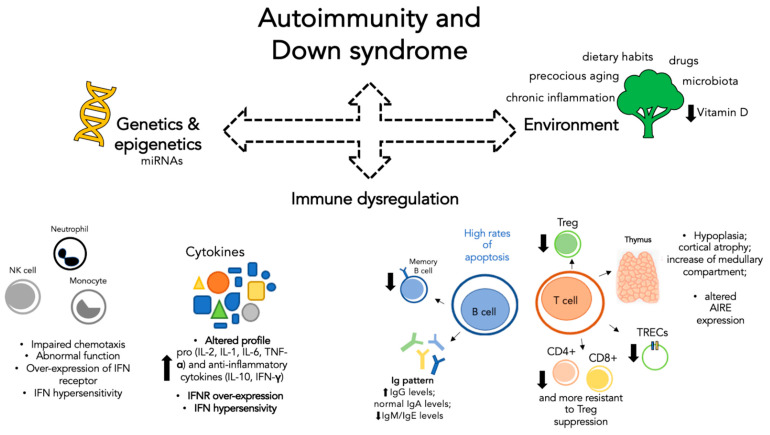
Schematic representation of factors contributing to autoimmunity in patients with Down syndrome. Autoimmunity represents a complex process in which immune dysregulation, genetics, and environmental factors act together. Each factor is capable of influencing and being influenced by the others. Immune dysregulation: Both the innate component (left) and the adaptive component (right) show alterations. Genetic and epigenetic: Genes and miRNAs. Environment: Precocious aging, dietary habits, drugs, microbiota, and chronic inflammation. (IFN: interferon; IFNR: interferon receptor; Ig: immunoglobulin; Treg: T regulatory cell; TRECs: T cell receptor excision circles; *AIRE*: autoimmune regulator.)

In general, the mechanisms underlying autoimmunity are extremely complex and immune dysregulation represents only one of the many faces of the problem. There are several environmental factors, not necessarily related to DS, that can influence the onset of autoimmune processes acting in concert with other factors, as shown in Figure 1. In recent years, for example, great attention has been paid to dietary habits and the health of the microbiota. Considered as a real organ, the intestinal microbiota plays a crucial role in the host’s immunity and is often altered during autoimmune diseases and chronic inflammation [31]. It is becoming evident that good dietary habits are essential not only to avoid obesity but also to ensure the health of the microbiota and thus reduce the risk of immune dysregulation. This topic plays a key role in patients with DS in whom early onset obesity and overweight represent commonly described comorbidities: poor dietary habits, low physical activities, and dyslipidemia are all capable of inducing microbiota modifications that predispose to a future autoimmunity process [32]. Correct dietary habits are also essential to ensure adequate intake of vitamins, such as vitamin D, for the proper functioning of the immune system. In this regard, it has been shown that compared to the general population, children with DS have lower vitamin D values. Possible causes of this phenomenon may be the lack of physical activity that often accompanies these patients in relation to cognitive impairment, less exposure to sunlight, and even reduced dietary intake [33]. Another environmental factor partly related to previous aspects is represented by drug therapies. Some drugs are able to predispose an individual to autoimmunity either by inducing alteration of microbiota, such as antibiotics, or by inducing the production of specific autoantibodies [34]. Patients with DS are more at risk because, due to recurrent infections and cardiac diseases, they frequently need prolonged therapies. Therefore, as shown in Figure 1, there is not only one factor that predisposes a person to autoimmunity but rather a set of environmental, genetic, and immune factors that combined in different ways can help define the susceptibility to autoimmunity. Additional studies are needed not only to identify other factors but also to understand how known factors may explain the phenotypic variability of these patients.

## 4. Down Syndrome: Autoimmunity Predisposition

In the wide spectrum of organ-specific autoimmune disorders that can involve patients with DS, some are more frequent than others. One of the most typical clinical features of these patients is their susceptibility toward several autoimmune diseases, such as Hashimoto’s thyroiditis (HT), Graves’ disease (GD), type 1 diabetes (T1D), celiac disease (CD), alopecia, vitiligo, and idiopathic arthritis [35]. Furthermore, the vast majority of clinical manifestations occur in the first decades of life, making early identification a key point in the fight against these diseases.

The risk of developing CD is increased 6- to 10-fold for DS patients compared to the general population and affects up to 7% of the patients [14,29,35]. Despite its frequency, diagnosis in people with DS can be very challenging. In a recent study by Liu et al., it was estimated that nearly half of the children with celiac disease did not have recognized symptoms, leading to a possibility of missed or delayed diagnoses. Furthermore, many of the known classic symptoms of onset, such as constipation, short stature, and behavior problems, can occur more frequently among children with DS independently of celiac disease. Altogether, these problems can cause a delay in diagnosis, estimated for this study, of three years [36]. Despite the high prevalence and the difficulties in diagnosis, there is no agreement for screening guidelines, specifically on when to start. The European Societies for Pediatric Gastroenterology, Hepatology, and Nutrition recommend screening all children with Down syndrome after age 3 years, and earlier if symptoms are apparent. The American Academy of Pediatrics (AAP) recommends only symptom-based screening from age one. This evidence, together with the complication associated with non-treated CD, reinforces the need for common guidelines for screening. 

As in the case of CD, an increased association between DS and the onset of diabetes mellitus has long been known. Specifically, subjects with DS have a 3- to 4-fold increased risk of developing T1D, which occurs more often at a younger age for them compared with the general population [29,36].

Although the association has long been observed, few studies have attempted to characterize diabetes in these patients, especially with regard to correlation with at-risk HLA (Human Leukocyte Antigens) haplotypes. In fact, it remains to be clarified whether subjects with DS are more prone to developing T1D due to the presence of predisposing HLA or due to the presence of an immune system predisposed to autoimmunity. 

This question arises after analyzing prevalence data of T1D in patients with DS in which it was shown that children with T1D and DS were less likely to carry the highest-risk genotype DR4-DQ8/DR3-DQ2 than children with T1D from the general population. A possible explanation can be searched in the immune dysregulation that characterizes the patients with DS, since they display a state of chronic inflammation characterized by IFN overexpression and hyperactivation [24]. Interestingly, a type 1 interferon transcriptional signature has been shown to precede autoimmunity in children genetically at risk of T1D [37]. Several other environmental aspects have been considered in the attempt to explain the association between T1D and DS: low birth weight, higher risk of infections, and different microbiome development represent only a few of them. Many questions remain unsolved, and further studies need to be done but it is probable that interferon hyperactivity plays a role in turning the immune system toward autoimmunity even in young children with DS [37]. 

Certainly, a chapter must be dedicated to thyroid since autoimmune hypothyroidism represents the most frequent autoimmune disorder in DS [1]. The association between thyroid autoimmunity and DS has long been known [6], and altered function of this organ represents the most common endocrinopathy seen in patients with this syndrome, with a percentage ranging from 7% to 66% [1]. Mild or subclinical hypothyroidism represents the main biochemical manifestation: unfortunately, clinical signs such as intellectual disability, skin dryness, and constipation are often characteristic of the syndrome and, therefore, complicate the diagnosis [38,39]. Among the various thyroid diseases, Hashimoto thyroiditis (HT) is, by far, the most common, with prevalence of 13–34% versus 1.3% in healthy subjects, followed by Graves’ disease (GD), with prevalence six times higher than the general pediatric population. Interestingly, both HT and GD debut at a younger age and do not present the known characteristics, such as prevalence in the female gender and positive family history. Furthermore, limited evidence suggests a higher probability of a spontaneous progression from HT to GD in children with DS [35]. When referring to HT, subclinical hypothyroidism is the most common biochemical pattern, frequently related to autoantibody formation (i.e., thyroid peroxidase (TPO) antibodies) and structural changes in the thyroid. In adults, up to 50% of individuals with Down syndrome have decreased thyroid function, and continued screening of thyroid stimulating hormone (TSH) and free T4 is advised at all ages [13]. On the contrary, with regard to GD, it appears as though there are no substantial differences among patients of the same age. Surprisingly, it seems that patients with DS with GD have a better response to the first cycle of methimazole and a lower frequency of relapses [35].

## 5. Conclusions

Down syndrome represents the genetic syndrome commonly associated with immune dysregulation [7]. In recent decades, the increase in life expectancy has led research to focus more on long-term complications such as increased risk of infections and autoimmunity that can reduce the quality of life of these patients. Genetic, transcriptomic, and proteomic studies are providing increasing insights for better understanding of these diseases. The genes contained in the additional chromosome 21 are not just a trivial extra copy but seem increasingly to play a more complex role in terms of hypo- or hyper-regulation of other elements through epigenetic modifications whose precise mechanisms have yet to be revealed. Immune dysregulation plays a crucial role in predisposing an individual toward autoimmunity. Concerning this field, the scenario is extremely complex, with a lack of agreement as to whether the immune system in DS is just intrinsically deficient or is another consequence of a generalized process of precocious aging. Several alterations of the immune system concerning both the innate and adaptive immunity compartments have been considered as possible underlying causes of autoimmunity. Certainly, the study of interferon hyperactivity and hypersensitivity will constitute an important field of research, especially with regard to the development of potential therapeutic targets. In addition to these known aspects, there are other factors not directly related to the chromosomal asset or to the immune dysregulation that may probably modify the onset and subsequent course of autoimmunity that still need to be investigated. It is likely that the key to understanding the autoimmunity process in DS patients will come from a comprehensive analysis of all contributing factors, genetics, immunity, and environment, and how they affect each other. 

## Data Availability

Not applicable.
